# Evaluation of Cochlear Implant Candidates using a Non-linguistic Spectrotemporal Modulation Detection Test

**DOI:** 10.1038/srep35235

**Published:** 2016-10-12

**Authors:** Ji Eun Choi, Sung Hwa Hong, Jong Ho Won, Hee-Sung Park, Young Sang Cho, Won-Ho Chung, Yang-Sun Cho, Il Joon Moon

**Affiliations:** 1 Department of Otorhinolaryngology - Head and Neck Surgery, Samsung Medical Center, Sungkyunkwan University School of Medicine, Seoul, Republic of Korea; 2 Department of Otorhinolaryngology - Head and Neck Surgery, Samsung Changwon Hospital, Sungkyunkwan University School of Medicine, Seoul, Republic of Korea; 3 Division of Ophthalmic and Ear, Nose and Throat Devices, Office of Device Evaluation, Center for Devices and Radiological Health, US Food and Drug Administration, Silver Spring 20993, Maryland, USA

## Abstract

Adults who score 50% correct or less in open-set sentence recognition test under the best aided listening condition may be considered as candidates for cochlear implant (CI). However, the requirement for ‘the best aided listening condition’ needs significant time and clinical resources to ensure such condition. As speech signals are composed of dynamic spectral and temporal modulations, psychoacoustic sensitivity to the combinations of spectral and temporal modulation cues may be a strong predictor for aided speech recognition. In this study, we tested 27 adults with moderately severe to profound hearing loss to explore the possibility that a non-linguistic unaided spectrotemporal modulation (STM) detection test might be a viable option as a surrogate measure to evaluate CI candidacy. Our results showed that STM detection thresholds were significantly correlated with aided sentence recognition scores for the 27 hearing impaired listeners. The receiver operator characteristic (ROC) curve analysis demonstrated that the CI candidacy evaluation by both unaided STM detection test and the traditional best-aided sentence recognition test was fairly consistent. More specifically, our results demonstrated that the STM detection test using a low spectral and temporal modulation rate might provide an efficient process for CI candidacy evaluation.

Cochlear implant (CI) is a surgically implanted auditory prosthesis that can provide successful speech recognition for individuals who have severe to profound bilateral sensorineural hearing loss. CIs consist of two components: (1) an external device such as microphones, speech processor, and transmitter system, and (2) a surgically implanted receiver/stimulator and an electrode array. When the microphone receives an acoustic sound from an outside environment, it sends the sound to the speech processor. The sound is then analyzed and converted into electrical signals. The speech processor then sends electric pulse trains to the electrode array in the cochlea which stimulates auditory nerves. The resulting neural signals then travel through the central nervous system to the brain and produce a sensation of hearing. Children and adults with severe to profound sensorineural hearing loss who receive little or no benefit from hearing aids (HAs) are considered as candidates for cochlear implantation.

Currently, there are three approved CI systems (Clarion, Nucleus, and MED-EL) in South Korea. CI candidacy criteria have evolved over time due to advances in CI technology and clinical investigation on the system’s safety and efficacy. Current guidelines in Korea permit cochlear implantation in children 12 to 23 months of age with profound hearing loss (i.e., pure tone average thresholds of 90 dB HL or greater) and in patients age 2 years and older with severe to profound hearing loss (i.e., pure tone average thresholds of 70 dB HL or greater). Speech perception tests are the most decisive ones in determining the candidacy of cochlear implantation. Adults who score 50% correct or less in open-set sentence recognition may be considered as candidates for CI. Audiological evaluation includes an assessment of current amplification. For example, the Korean National Health Insurance recommends three to six months of hearing aid trial for children. Appropriately fitted binaural hearing aids may be needed for adults before speech testing. Limited benefit from hearing aids is defined by speech perception test scores under the best aided listening condition.

However, the current guidelines require significant time and effort to ensure the best aided listening condition. Shim *et al.* (2014) have explored the possibility of using surrogate non-speech psychoacoustic measures without HA to address this limitation^1^. Shim *et al.* have assessed unaided spectral ripple discrimination (SRD) and temporal modulation detection in fifteen hearing impaired listeners and compared their performances on psychoacoustic tasks with aided speech perception tasks^1^. Significant correlations between aided speech perception outcomes and unaided spectral modulation sensitivity as well as temporal modulation detection performance have been reported. Most importantly, Shim *et al.* have demonstrated that the unaided SRD test could be a promising tool for evaluating CI candidacy^1^.

Although speech is composed of dynamic spectral and temporal modulations that can change depending on utterance, previous studies have often measured spectral or temporal modulation sensitivities separately. It is possible that a combination of spectral and temporal modulation cues can be delivered as test signals^2^,^3^. These stimuli, often called “spectrotemporal modulation (STM)”, represent spectral patterns that can change over time or temporal modulation patterns that can differ across frequency channels. Bernstein *et al.* have reported that STM sensitivity, especially those with low-rate but high-density stimuli, is strongly correlated with speech intelligibility for hearing impaired listeners^4^. However, no studies have evaluated unaided STM sensitivity as a predictor for speech perception performance in hearing impaired listeners with severe to profound sensorineural hearing loss who might be potential CI candidates^4^,^5^. Won *et al.* have recently demonstrated that STM detection thresholds for low spectral densities are significantly correlated with sentence identification scores in CI users. Based on the results of these previous studies, we hypothesized that the unaided STM detection test might serve as a viable surrogate measure for CI candidacy evaluation. To test this hypothesis, the performance of the unaided STM test was determined and compared to speech perception performance measured under the best aided listening condition.

## Results

### Speech perception and psychoacoustic performance

**Figure 1:**
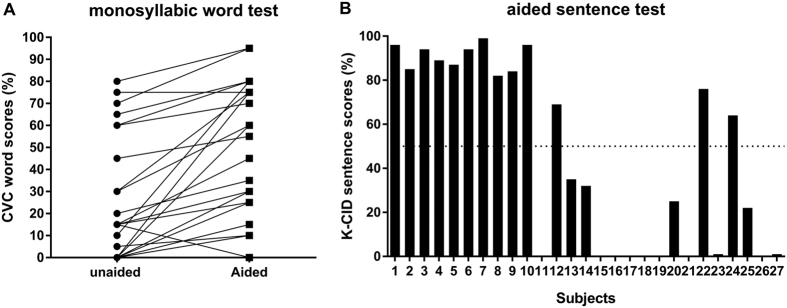
Speech perception performance for each subject. (**A**) Monosyllabic word recognition tests in a quiet background for each subject were performed without HAs, with their own HAs, or loaner HAs. Unaided monosyllabic word recognition scores ranged from 0% to 80%. Aided scores ranged from 0% to 95%. (**B**) The average of two list sentence recognition scores in “best-fit” condition ranged from 0% to 99%. Dotted line indicates 50% correct scores. HAs: hearing aids, CVC: consonant-vowel-consonant, K-CID: Korean Central Institute for the Deaf.

**Figure 2:**
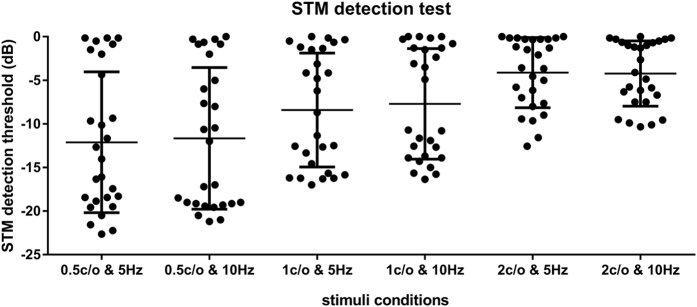
Unaided spectrotemporal modulation (STM) detection performance for each subject. Circle (•) indicates STM detection threshold (dB) for each stimuli condition. Mean STM detection thresholds at 0.5 c/o & 5 Hz, 0.5 c/o & 10 Hz, 1 c/o & 5 Hz, 1 c/o & 10 Hz, 2 c/o & 5 Hz, and 2 c/o & 10 Hz were −11.6 dB, −12.3 dB, −9.3 dB, −6.8 dB, −4.6 dB, and −4.7 dB, respectively.

Speech perception performances for twenty-seven hearing impaired participants are shown in Fig. 1. Unaided monosyllabic word recognition scores ranged from 0% to 80% (mean scores: 23%). Aided monosyllabic word recognition scores ranged from 0% to 95% (mean scores: 42%) (Fig. 1A). Aided sentence recognition scores for each subject ranged from 0% to 99% (mean scores: 46%) (Fig. 1B). Speech recognition abilities varied substantially across subjects. The performance results of STM detection without HAs are shown in Fig. 2. Here, lower STM detection threshold indicated better detection performance. When the thresholds for spectral densities of 0.5, 1.0, and 2.0 c/o were compared to each other, subjects performed better at lower spectral densities. Across different stimulus conditions, substantial variabilities in STM thresholds across subjects were found.

### Correlation analyses

**Figure 3:**
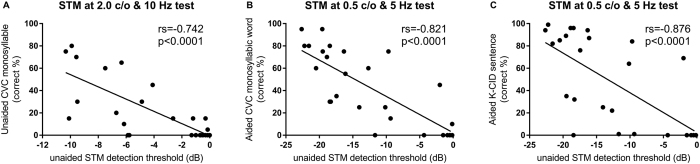
Scattergram of spectrotemporal modulation (STM) detection tests and speech recognition tests. X-axis represents unaided STM detection test while Y-axis represents speech recognition scores. Panel A indicates unaided CVC monosyllabic word scores. Panel B indicates aided CVC monosyllabic word scores. Panel C indicates aided K-CID sentence scores. Among stimulus conditions of STM detection test, scattergam was presented only for stimuli condition that showed the best correlation with speech recognition scores. rs, Spearman correlation coefficient; p, significance.

Results of correlation between unaided STM detection thresholds and speech recognition scores are summarized in Table 1. Significant correlations were found between all STM detection thresholds and the three speech recognition scores for the 27 participants. STM detection thresholds at 2.0 c/o & 10 Hz showed the best correlation with unaided monosyllabic consonant-vowel-consonant (CVC) word scores (r = −0.742, *p* < 0.0001). Under the aided condition, STM detection thresholds at 0.5 c/o & 5 Hz significantly correlated with monosyllabic CVC word scores (r = −0.821, *p* < 0.0001) and K-CID sentence recognition scores (r = −0.762, *p* < 0.0001). The scattergram of unaided STM tests and aided speech perception scores is shown in Fig. 3.

### ROC Curve Analysis

To further investigate the clinical potential of the unaided STM detection test as a surrogate measure of CI candidacy evaluation, a receiver operator characteristic (ROC) curve analysis was used to assess the quality of CI candidate evaluation by STM tests. The optimal cutoffs of psychoacoustic measures were determined by finding the values for predicting K-CID sentence recognition scores less than 50%.

**Figure 4:**
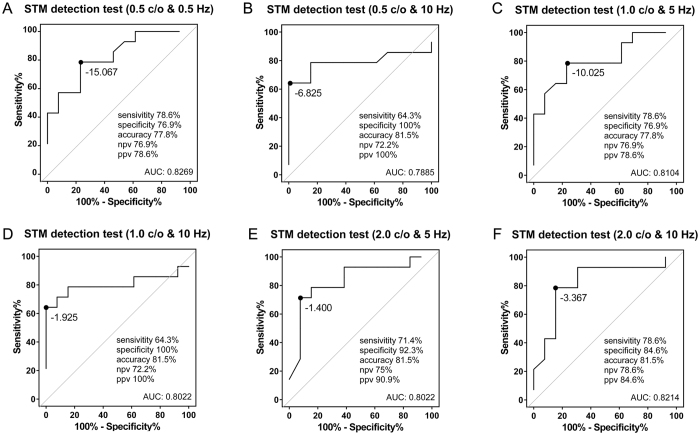
ROC curves in spectrotemporal modulation (STM) detection tests. (**A**–**G**) ROC curves for 27 ears are plotted for CI candidacy evaluation using STM detection thresholds at 0.5 c/o & 5 Hz (**A**), 0.5 c/o & 10 Hz (**B**), 1.0 c/o & 5 Hz (**C**), 1.0 c/o & 10 Hz (**D**), 2.0 c/o & 5 Hz (**E**), and 2.0 c/o & 10 Hz (**F**). npv, negative predictive value; ppn, positive predictive value; AUC, area under the curve.

The ROC curves for the 27 ears in STM detection tests at six stimulus conditions are shown in Fig. 4A to F using sensitivity (%) as a function of specificity (100% - specificity). The areas under the ROC curve in Fig. 4A–F were 0.83, 0.79, 0.81, 0.80, 0.80, and 0.82, respectively. The optimal cutoff values for the STM detection performance at 0.5 c/o & 5 Hz, 0.5 c/o & 10 Hz, 1.0 c/o & 5 Hz, 1.0 c/o & 10 Hz, 2.0 c/o & 5 Hz, and 2.0 c/o & 10 Hz were −15.067 dB, −6.825 dB, −10.025 dB, −1.925 dB, −1.400 dB, and −3.367 dB, respectively. Subjects showing worse STM thresholds than these cutoff values would be designated as CI candidates. For STM detection performance at 0.5 c/o & 5 Hz which showed the strongest correlation with K-CID sentence scores, the corresponding optimized sensitivity value, specificity, positive predictive value, negative predictive value, and accuracy were 78.6%, 76.9%, 78.6%, 76.9%, and 77.8%, respectively, at the optimal cutoff value (Fig. 4A). For STM detection performance at 2.0 c/o & 5 Hz which showed the second strongest correlation with K-CID sentence scores with the highest accuracy value at optimal cutoff thresholds, the sensitivity, specificity, positive predictive value, negative predictive value, and accuracy were 71.4%, 92.3%, 90.9%, 75%, and 81.5%, respectively (Fig. 4E). For STM detection performance at 2.0 c/o & 10 Hz which also showed strong correlation with K-CID sentence scores with the highest accuracy value at optimal cutoff thresholds, the sensitivity, specificity, positive predictive value, negative predictive value, and accuracy were 78.6%, 84.6%, 84.6%, 78.6%, and 81.5%, respectively (Fig. 4F).

## Discussion

The current study evaluated the potential implication of unaided non-linguistic STM tests as surrogate measures for CI candidacy evaluation. Shim *et al.* have demonstrated that unaided spectral-ripple discrimination test could be used a promising tool for evaluating CI candidacy^1^. Although their data were not reported in this paper, we have successfully replicated the finding of Shim *et al.*^1^ using 25 of the 27 patients who participated in the current study, underscoring the efficacy of a non-linguistic psychoacoustic test as an efficient surrogate measure under any language environment.

While previous studies have often measured spectral or temporal modulation sensitivities separately, the STM detection test in this study used test signal as a combination of spectral and temporal modulation cues for evaluating the potential CI candidacy. Unaided STM thresholds showed significant correlations with all three speech recognition tests. Among six stimulus conditions for the STM detection test, STM thresholds at 0.5 c/o & 5 Hz showed the strongest correlation with K-CID sentence scores (r = −0.762, *p* < 0.0001). Anderson *et al.* and Saoki *et al.* have demonstrated that spectral modulation detection thresholds at lower spectral densities (0.25 to 0.5 c/o) have stronger correlations with speech perception abilities in CI users^6^,^7^, consistent with the finding of the current study. STM thresholds at 0.5 c/o & 5 Hz also correlated significantly with aided CVC word scores (r = −0.821, *p* < 0.0001) and aided K-CID sentence scores (r = −0.876, *p* < 0.0001) in this study. In general, unaided STM detection thresholds correlated with aided speech performances more than with unaided monosyllabic CVC word recognition scores (Table 1).

Because cochlear implantation is typically approved for patients with limited benefit from appropriately fit HAs (defined by sentence recognition score), ROC curve analysis was performed between STM detection test scores and K-CID sentence scores. Results of the ROC curve analysis demonstrated that the STM detection test using low spectral and temporal modulation rates (0.5 c/o & 5 Hz) may serve as a good complementary measure for evaluating CI candidacy with a derived area under the ROC curve of 0.8269. Based on speech perception performances and results of the CI candidacy prediction made by the STM tests (Table 2), among subjects who were predicted to meet CI candidacy by all STM detection tests, none showed a sentence score of more than 50% under the best aided condition. When the prediction of CI candidacy based on cutoff values of six STM detection thresholds was compared to that based on K-CDI sentence scores, only three subjects (S11, S13, and S14) failed to show consistent CI candidacy prediction between sentence recognition test and STM detection tests. Although S11, S13, and 14 showed sentence scores of less than 50% under the best aided condition, three subjects had relatively good hearing at least on one side (See Tables 2 and 3).

Aided speech perception test requires significant resource investment such as time and effort to ensure the best aided listening condition. In addition, unnecessary costs could incur if HA users receive cochlear implantation. Thus, unaided psychoacoustic measures could be cost-effective because these tests can be implemented without having to fit HAs. The STM test in the current form took about a total of 60 minutes for the six stimuli conditions. However, the testing time could be reduced by optimizing stimulus conditions that could potentially show the best prediction power such as by using STM stimulus conditions with spectral density of 0.5 c/o and temporal rate of 5 Hz or 10 Hz. Such efforts have already been reported for the development of clinical assessment of psychoacoustic performance^8^,^9^. Furthermore, STM test tasks can be readily performed within any language system because they use non-speech stimuli. Consequently, they are less likely to be influenced by other non-auditory factors that might affect speech recognition such as patient’s cognitive processing ability, educational background, and age^1^,^10^. In addition, it might be used to create an international or cross language standard for CI candidacy with psychoacoustic measures. In addition, no learning effect was observed in psychoacoustic test. However, speech perception test has a chance of having learning effect due to limited number of sentences in the test list if listeners are repeated with the same test material^10^. Although it may be premature to use the STM test as the gold standard CI candidacy measure, the current study demonstrates that the STM detection test may be a useful complementary measure to determine the CI candidacy. Further studies are needed to optimize the STM stimulus conditions and testing paradigm to improve its CI candidacy evaluation power.

## Conclusion

The current study showed strong correlations between speech intelligibility and STM detection thresholds, especially for STM test at spectral density of 0.5 c/o and temporal modulation rate of 5 Hz. The CI candidacy prediction based on the STM detection test demonstrated good accuracy for evaluating CI candidacy compared to sentence recognition scores under the best aided condition. Results from the complementary STM test may provide clinicians more accurate prediction of CI candidacy. Unaided psychoacoustic measure may be particularly appealing to clinicians when resources for HA trials are limited.

## Methods

### Subjects

**Figure 5:**
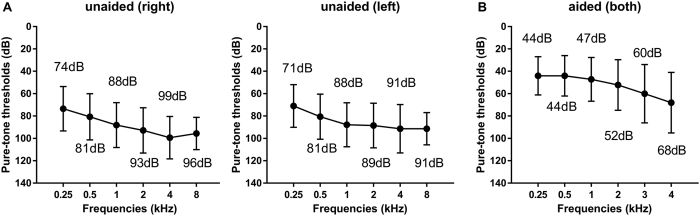
Mean audiograms of each subject. (**A**) Mean audiogram (±2 SD) for each listener without HAs at octave frequencies between 0.25 kHz and 8 kHz. (**B**) Mean audiogram (±2 SD) for each listener under best-fit aided listening condition at frequencies between 0.24 kHz and 4 kHz. Subjects used loaner HAs if they did not have their own HAs. HAs: Hearing aids.

Twenty-seven hearing impaired listeners participated in this study. All subjects were native Korean speaking adults with hearing loss greater than 56 dB HL in both ears on average at four frequencies (0.5 Hz, 1 kHz, 2 kHz, and 4 kHz). Their mean auditory performances at each frequency are shown in Fig. 5. Demographic characteristics of these subjects are shown in Table 4. All participants provided their written informed consent to participate in this study. The study protocol was approved Samsung Medical Center Institutional Review Board (2013-06-031). This study was carried out in accordance with approved guidelines.

### Test battery administration

All subjects participated in all STM detection and speech recognition tests. A custom made MATLAB^®^ (The Mathworks, Natick) graphical user interface was used to present acoustic stimuli to subjects for psychoacoustic tests. Stimuli were generally presented at the most-comfortable level (MCL) which was determined after frequency-dependent amplification using the half-gain rule. Briefly, the stimuli were first set at 65 dB SPL. A frequency of independent gain equal to half of the pure-tone average was then applied to the stimuli. Finally, the presentation levels of the stimuli were adjusted within a range of ±10 dB to estimate the most-comfortable level for individual participants. Pure-tone thresholds and MCLs for all tested ears are summarized in Table 3. The amplified stimuli were then presented binaurally through inserted ear phones. Speech recognition tests with a quiet background were conducted using monosyllabic words and sentences with or without HAs. Aided speech recognition tests were conducted under the best-fit listening condition using participants’ own HAs or loaner HAs if they did not have their own. The order of test administration varied within and across subjects.

### Spectrotemporal Modulation (STM) Detection Test

To create STM stimuli with a bandwidth of four octaves (i.e., 354–5664 Hz), the following equation was used based on previously established technique^2^:


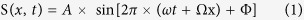


**Figure 6:**
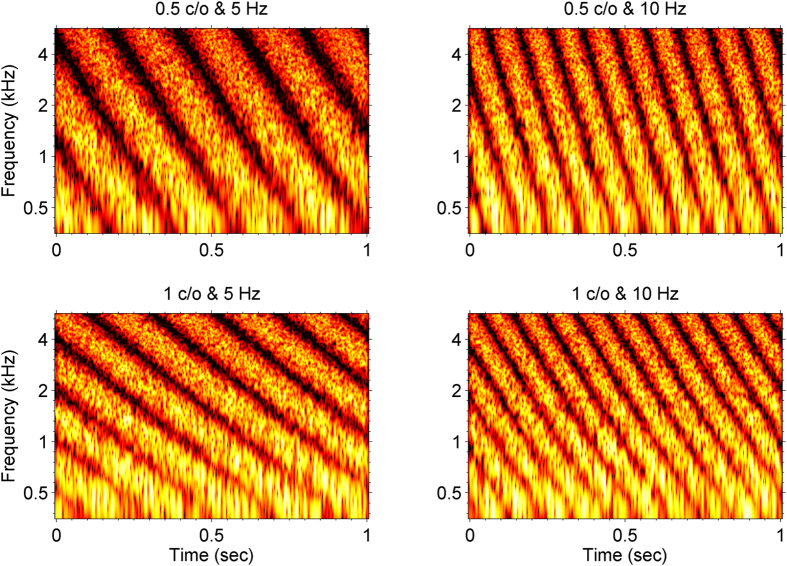
Example of spectrograms for spectrotemporal modulation (STM) stimuli with four different combinations of spectral density and temporal rate. In these spectrograms, different amplitudes of STM stimuli are depicted as color intensities over a dynamic range of 40 dB.

In Eq. (1), *S* ws the amplitude of each carrier tone as a function of time (*t*) and logarithmic frequency (*x*) (i.e., x = log_2_(*f*/354), where *f* was the frequency). Four thousands carrier tones were spaced equally on a logarithmic frequency scale with bandwidth of 354–5656 Hz. The stimuli had a total duration of 1 sec. The spectral envelope of complex tones was modulated as a single sinusoid along the logarithmic frequency axis on a linear amplitude scale. The amplitude (A) of the rippled spectral modulation was determined by adaptively adjusting the modulation depth of all carrier tones simultaneously. When A was set to a value between 0 and 1, it corresponded to 0 to 100% spectral modulation of the flat ripple envelope. Ω was the spectral density in units of cycles per octave (*c/o*). Φ was the spectral modulation starting phase in radians for carrier tones randomized in radians (range, 0 to 2π). The STM stimuli were also modulated in time by having modulated spectral envelopes sweep across the frequency at a constant velocity. In Eq. (1), ω was the spectral modulation velocity expressed as the number of sweeps per second (Hz). This was referred to as temporal rate in the current study. The positive and negative velocity constructed the STM stimuli with spectral modulations (or frequency modulations) that either increased or decreased in frequency and repeated over time. Since a previous study has shown that the direction of spectral modulation has no effect on STM detection thresholds for normal hearing or hearing impaired listeners^4^, the current study tested a falling direction of spectral modulation alone. Figure 6 shows an example of spectrograms of STM stimuli with different combinations of spectral density and temporal rate. The upper and lower rows are spectrograms for STM stimuli with a spectral density of 0.5 and 1.0 cycle per octave (c/o), respectively. As shown in Fig. 6, there is a relatively broader spectral modulation pattern in the upper row along the frequency domain than that in the lower row. The spectrograms for STM stimuli with a temporal rate of 5 and 10 Hz are shown in the left column and the right column, respectively. The temporal rate determines the speed of frequency sweep that falls from high to low frequency along the frequency domain.

To measure STM detection thresholds, a 2-interval, 2-alternative adaptive forced-choice (2I, 2-AFC) paradigm was used. A silence interval of 500 ms was used between the two intervals. One interval consisted of modulated noise (i.e., test signal) while the other interval consisted of steady noise (i.e., reference signal). Subjects were instructed to choose an interval containing sound such as bird-chirping, vibrating, or moving over time and frequency. Subject’s task was to identify the interval that contained a STM stimulus. A 1-down, 1-up adaptive procedure was used to measure STM detection threshold starting with a modulation depth of 0 dB which was decreased in steps of 4 dB from the first to the fourth reversal and 2 dB for the next 10 reversals. For each testing run, the final 10 reversals were averaged to obtain the STM detection threshold. In order to evaluate STM detection performance at different modulation conditions, three different spectral densities (Ω = 0.5, 1, and 2 c/o) and two different temporal rates (ω = 5 and 10 Hz) were tested. Thus, a total of six different sets of STM stimuli were tested. Subjects completed the tests under the six different stimulus conditions in a random order. Subjects then repeated a new set of tests under the six stimulus conditions in a newly created random order. The sequence of stimulus conditions was randomized within and across subjects. A third adaptive track was obtained if the difference between the first two tracks exceeded 3 dB for a given stimulus condition. The final threshold for each STM stimulus condition was the mean of two (or three) adaptive tracks. Before actual testing, example stimuli were played for subjects until they became familiar with STM stimuli and the task. The entire procedure (including rehearsal and actual testing for all stimulus conditions) took about 60 minutes to complete.

### Speech recognition tests in quiet background

Two different types of speech recognition tests were administered in the current study using either monosyllabic words or sentences. For monosyllabic word recognition test, twenty mono-syllabic consonant-vowel-consonant (CVC) words were presented with live male voice at individual subject’s most comfortable level (MCL). For unaided monosyllabic word test, stimuli were presented at MCL which was determined after frequency-dependent amplification using the half-gain rule (Table 3). For aided monosyllabic word test, stimuli were presented at a calibrated level of 65 dB HL. A total percent correct score was calculated based on words that were correctly repeated. For the sentence recognition test, two lists of Korean Central Institute for the Deaf (K-CID) sentences were administered at an average level of 65 dBA^11^. Each list contained ten sentences with four keywords. Therefore, a total of 80 keywords were scored for each subject. All participants were instructed to verbally repeat the sentence that they heard. A total percent correct score was calculated as the percent of keywords correctly recognized.

Monosyllabic word recognition test was performed with or without HA. For aided testing, subjects used either their own HAs or laboratory-owned HAs (Audeo S Smart V, Phonak, Switzerland). Sentence recognition test was always administered with HAs. Unaided monosyllabic word recognition test was presented via a headphone (TDH39, Telephonics, USA). When performing aided monosyllabic word or sentence recognition test, speech stimuli were presented through a loud speaker (Control 1 Xtream, JBL, USA for monosyllabic word test and HS-50M, Yamaha, Japan for sentence recognition test) in a sound-field. Subjects sat one meter away from the loudspeaker. They were asked to face it during the course of the experiment.

### Analysis

To estimate the optimal psychoacoustic criterion value for CI candidacy, exploratory analysis was performed using Spearman correlation analysis and ROC curve analysis. ROC curve was plotted using measures of sensitivity and specificity based on various anthropometric cutoff values to determine the optimal cutoff value for predicting CI candidacy. Statistical analyses were performed using SAS version 9.4 (SAS Institute, Cary, NC) and R 3.2.1 (Vienna, Austria; http://www.R-project.org/). Bonferroni corrections were not applied due to the increased risk of a type II error for the number of comparisons made (e.g., Benjamini Y., Hochberg Y. (1995), cited in Hughes and Stille, 2010; Won *et al.*, 2014; Won *et al.* 2015)^10^,^12^,^13^,^14^. Instead, correlation coefficients and their associated *p*-values were provided.

## Additional Information

**How to cite this article**: Choi, J. E. *et al.* Evaluation of Cochlear Implant Candidates using a Non-linguistic Spectrotemporal Modulation Detection Test. *Sci. Rep.* **6**, 35235; doi: 10.1038/srep35235 (2016).

## Figures and Tables

**Table 1:** Correlations between spectrotemporal modulation (STM) detection tests and speech recognition performances.

Stimuli conditions	STM detection tests											
	0.5 c/o & 5 Hz		0.5 c/o & 10 Hz		1.0 c/o & 5 Hz		1.0 c/o & 10 Hz		2.0 c/o & 5 Hz		2.0 c/o & 10 Hz	
	rs	*P*	rs	*P*	rs	*P*	rs	*P*	rs	*P*	rs	*P*
Unaided CVC word	−0.617	0.001	−0.517	0.006	−0.645	<0.0001	−0.585	0.001	−0.711	<0.0001	−0.742	<0.0001
Aided CVC word	−0.821	<0.0001	−0.719	<0.0001	−0.778	<0.0001	−0.743	<0.0001	−0.713	<0.0001	−0.750	<0.0001
Aided K-CID sentence	−0.762	<0.0001	−0.632	<0.0001	−0.655	<0.0001	−0.633	<0.0001	−0.717	<0.0001	−0.704	<0.0001

Correlations between STM detection tests and unaided CVC monosyllabic word test, aided CVC monosyllabic word test, or sentence recognition in quiet (K-CID) in hearing impaired subjects were determined. rs, Spearman correlation coefficient; p, significance. Bold values indicate significant correlations at the level of 0.05. CVC, consonant-vowel-consonant; K-CID, Korean Central Institute for the Deaf.

**Table 2:** Auditory performances and result of CI candidacy prediction based on spectrotemporal modulation (STM) detection thresholds.

Subject	K-CID sentence (%)	Unaided PTA*		STM detection tests					
		Right	Left	0.5 c/o & 5 Hz	0.5 c/o & 10 Hz	1.0 c/o & 5 Hz	1.0 c/o & 10 Hz	2.0 c/o & 5 Hz	2.0 c/o & 10 Hz
S1	96	88.8	71.3	No	No	No	No	No	No
S2	85	103.8	62.5	No	No	No	No	No	No
S3	94	61.3	58.8	No	No	No	No	No	No
S4	89	63.8	83.8	No	No	No	No	No	No
S5	87	86.3	82.5	No	No	No	No	No	No
S6	94	81.3	76.3	No	No	No	No	No	No
S7	99	71.3	70.0	No	No	No	No	No	No
S8	82	67.5	62.5	No	No	No	No	No	No
S9	84	70.0	70.0	Yes	No	Yes	No	Yes	No
S10	96	81.3	83.8	No	No	No	No	No	No
S11	0	105.0	71.3	No	No	No	No	No	No
S12	69	87.5	56.3	Yes	No	Yes	No	No	Yes
S13	35	77.5	81.3	No	No	No	No	No	No
S14	32	60.0	103.8	No	No	No	No	No	No
S15	0	113.8	91.3	Yes	Yes	Yes	Yes	Yes	Yes
S16	0	115.0	110.0	Yes	Yes	Yes	Yes	Yes	Yes
S17	0	108.8	108.8	Yes	Yes	Yes	Yes	Yes	Yes
S18	0	95.0	117.5	Yes	Yes	Yes	Yes	Yes	Yes
S19	0	106.3	105.0	Yes	Yes	Yes	Yes	Yes	Yes
S20	25	103.8	103.8	Yes	No	Yes	No	No	Yes
S21	0	118.8	117.5	Yes	Yes	Yes	Yes	Yes	Yes
S22	76	78.8	77.5	No	No	No	No	No	No
S23	1	85.0	85.0	Yes	Yes	Yes	No	Yes	Yes
S24	64	98.8	102.5	Yes	No	Yes	No	No	Yes
S25	22	85.0	83.8	Yes	Yes	Yes	Yes	Yes	Yes
S26	0	108.8	113.8	Yes	Yes	Yes	Yes	Yes	Yes
S27	1	113.8	100.0	Yes	No	Yes	Yes	Yes	Yes

CI, Cochlear Implant; K-CID, Korean Central Institute for the Deaf; PTA, pure tone average. PTA*, average threshold for four frequencies (0.5, 1 k, 2 k, and 4 k Hz).

**Table 3:** Pure-tone thresholds and MCLs measured for spectrotemporal modulation (STM) detection tests.

Subjects	Right ear					Left ear				
	Pure-tone thresholds				MCL	Pure-tone thresholds				MCL
	500	1K	2K	4K		500	1K	2K	4K	
S1	70	95	95	95	105	60	75	80	70	95
S2	90	105	105	115	101	65	60	60	65	88
S3	55	70	65	55	85	55	65	60	55	84
S4	60	65	65	65	86	90	85	75	85	96
S5	65	70	100	110	96	60	65	100	105	96
S6	80	80	75	90	80	75	75	70	85	80
S7	45	55	75	110	90	50	60	80	90	90
S8	60	65	70	75	80	60	70	60	60	80
S9	55	70	80	75	85	60	80	75	65	90
S10	65	70	75	115	92	70	70	75	120	92
S11	110	105	110	95	100	85	75	70	55	91
S12	90	80	85	95	97	45	55	60	65	83
S13	70	80	85	75	90	65	90	90	80	90
S14	60	55	55	70	85	105	105	100	105	106
S15	105	120	115	115	111	80	105	95	85	96
S16	115	115	115	115	113	105	115	110	110	110
S17	100	100	115	120	110	100	105	115	115	110
S18	60	80	120	120	103	120	120	115	115	113
S19	90	110	110	115	105	95	110	105	110	105
S20	85	110	110	110	110	80	105	115	115	110
S21	115	120	120	120	114	115	120	120	115	113
S22	75	75	70	95	88	80	85	70	75	88
S23	85	80	75	100	96	85	80	85	90	96
S24	70	95	115	115	106	80	100	115	115	106
S25	85	85	80	90	102	85	85	80	85	102
S26	115	110	105	105	115	110	115	115	115	115
S27	105	115	115	120	111	95	95	95	115	105

MCL, Most Comfortable Level.

**Table 4:** Demographic characteristics of subjects.

Subject	Sex	Age (yr)	Duration of hearing loss (yr)	Etiology	HA experience (yr)	
					Right	Left
S1	M	30	19.0	Unknown	N/A	4.2
S2	M	25	5.9	Unknown	N/A	3.8
S3	F	40	20.3	Unknown	14.3	5.0
S4	M	53	15.9	Right: Meniere	13.0	3.3
S5	M	58	12.0	Unknown	4.0	2.0
S6	F	80	30.0	COM	N/A	N/A
S7	M	53	10.1	Unknown	4.9	4.9
S8	M	46	8.4	Unknown	6.4	6.4
S9	F	72	29.1	NIHL	9.0	12.3
S10	M	71	15.4	Unknown	10.3	10.3
S11	M	31	11.4	Unknown	N/A	1.1
S12	M	72	8.0	Right: Unknown, Left: COM	N/A	N/A
S13	F	28	15.0	Unknown	5.5	13.5
S14	M	57	15.0	Right: SSNHL, Left: NIHL	N/A	N/A
S15	F	35	10.8	Ototoxic drug	N/A	10.4
S16	F	57	10.0	Unknown	7.0	7.0
S17	M	35	35.1	Unknown	1.0	1.0
S18	M	54	10.0	Unknown	N/A	N/A
S19	M	60	48.2	Unknown	20.0	20.0
S20	F	23	20.0	Unknown	18.0	18.0
S21	F	62	20.0	Unknown	15.0	N/A
S22	M	61	30.0	NIHL	0.4	N/A
S23	M	87	40.0	Right: Meniere	N/A	8.0
S24	F	52	30.0	Unknown	18	15
S25	F	19	14	Unknown	14	14
S26	F	34	5.4	Ototoxicity drug	2	N/A
S27	M	35	11.5	Unknown	0.1	11.3

COM, Chronic otitis media; NIHL, noise induced hearing loss; SSNHL, sudden sensorineural hearing loss; N/A, not applicable.
